# Are the High Serum Interleukin-6 and Vascular Endothelial Growth Factor Levels Useful Prognostic Markers in Aggressive Non-Hodgkin Lymphoma Patients?

**DOI:** 10.4274/tjh.2013.0325

**Published:** 2015-02-15

**Authors:** Hava Üsküdar Teke, Eren Gündüz, Olga Meltem Akay, Cengiz Bal, Zafer Gülbaş

**Affiliations:** 1 Osmangazi University Faculty of Medicine, Department of Hematology, Eskişehir, Turkey; 2 Osmangazi University Faculty of Medicine, Department of Biostatistics, Eskişehir, Turkey; 3 Anadolu Health Center, Bone Marrow Transplantation Center, Kocaeli, Turkey

**Keywords:** s-IL6, s-VEGF, Lymphoma, overall survival

## Abstract

**Objective:**

Pro-inflammatory and pro-angiogenic cytokines play an important role in the pathogenesis of lymphoma, and recent studies have shown that cytokines can be used as prognostic markers. Non-Hodgkin lymphoma (NHL) patients with high levels of serum interleukin-6 (s-IL6) and serum vascular endothelial growth factor (s-VEGF) have poor prognosis and shorter survival time. We aimed to determine pre-treatment levels of s-IL6 and s-VEGF and their relation with known prognostic markers, especially International Prognostic Index (IPI) scores, and to examine their effects on overall survival in newly diagnosed, untreated aggressive NHL patients.

**Materials and Methods:**

The study included 51 newly diagnosed NHL patients and 17 healthy controls. Blood samples were obtained to study s-IL6 and s-VEGF cytokine levels.

**Results:**

Patients with aggressive NHL diagnosis had higher s-VEGF and s-IL6 levels than the healthy population. If the s-IL6 levels of patients were above the cut-off levels, the overall survival time was shorter. There was no relation between s-VEGF and overall survival time.

**Conclusion:**

s-IL6 is an independent prognostic factor that may be included in IPI risk classification. In addition to the s-IL6 level, age, erythrocyte sedimentation rate, beta-2 microglobulin, WHO performance status, and IPI score are independent prognostic factors that are effective, especially for overall survival, in the clinical follow-up of NHL patients.

## Abstract (tr)

**Amaç:**

Pro-inflamatuvar ve pro-angiogenik sitokinlerin, lenfomanın patogenezinde önemli rolleri olduğu kadar son yıllarda yapılan çalışmalarda sitokinlerin prognostik birer belirteç olarak da kullanılabileceği gösterilmiştir. s-IL6 ve s-VEGF düzeyleri yüksek olan non-Hodgkin lenfoma (NHL) hastalarında prognoz daha kötüdür ve genel sağkalımları daha kısadır. Bu çalışmada yeni tanı almış, tedavi verilmemiş NHL hastalarında tedavi öncesi s-IL6 ve s-VEGF düzeylerinin belirlenmesi, bu sitokinlerin bilinen prognostik belirteçler ve özellikle International Prognostic Index (IPI) değerleri ile ilişkisinin saptanması ve genel sağkalım üzerine etkilerinin incelenmesi amaçlanmıştır.

**Gereç ve Yöntemler:**

Çalışmaya 51 yeni tanı konmuş NHL hastası ve 17 sağlıklı kontrol alındı. s-IL6 ve s-VEGF sitokin düzeylerini değerlendirmek için kan örnekleri toplandı.

**Bulgular:**

NHL ile izlenen hastalarda s-VEGF ve s-IL6 düzeyleri sağlıklı popülasyondan yüksektir. Hastaların özellikle s-IL6 düzeyleri cut-off değerin üzerinde ise genel sağkalımları kısa olmaktadır. s-VEGF ile genel sağkalım arasında ilişki saptanmamıştır.

**Sonuç:**

s-IL6, IPI risk sınıflamasına dahil edilebilir bağımsız bir prognostik faktördür. s-IL6 düzeyine ilave olarak, yaş, ESR, beta-2 mikroglobulin, WHO performans statüsü ve IPI skoru NHL hastalarının klinik takibinde özellikle genel sağkalım üzerine etkili olan bağımsız birer prognostik faktördür.

## INTRODUCTION

Various clinical and laboratory parameters were included in the prognostic definition of non-Hodgkin lymphoma (NHL) and are used in clinical follow-up. Pro-inflammatory and pro-angiogenic cytokines play an important role in the pathogenesis of lymphoma, and recent studies have shown that cytokines can be used as prognostic markers. Interleukin (IL)-6, which is one of these cytokines, is a lymphoid growth factor and is also an important protein for the immune system, hematopoiesis, and inflammation. It is responsible for the B symptoms in lymphoma [1,2,3]. Angiogenesis, the formation of new blood vessels, is required for the growth and spread of cancer cells [4]. Angiogenesis is regulated by a variety of positive and negative angiogenic molecules. The most capable and the most important angiogenic molecule is vascular endothelial growth factor (VEGF). Lymphoma tumor cells express VEGF and have been shown to promote survival, proliferation, and metastasis via autocrine mechanisms [5]. NHL patients with high levels of serum (s)-IL6 and s-VEGF have poor prognosis and short survival [6]. In addition, the pre-treatment s-IL6 and s-VEGF levels of NHL patients are correlated with life expectancy, and both of these are independent and important prognostic indicators for all International Prognostic Index (IPI) groups [6,7]. However, there is no information available for patients from the Turkish population.

In this study, we aimed to determine pre-treatment levels of s-IL6 and s-VEGF and their relation with known prognostic markers, especially IPI levels, and to examine their effects on overall survival (OS) in newly diagnosed, untreated aggressive NHL patients in a Turkish population.

## MATERIALS AND METHODS

### Patients and Controls

This study was performed between April 2006 and October 2007 in the Hematology Division of the Eskişehir Osmangazi University (ESOGU) Medical Faculty’s Internal Medicine Department. The approval of the ESOGU Medical Faculty Ethics Committee and informed consent from subjects, including all necessary explanations, were obtained for this study. The study included 51 newly diagnosed NHL patients. Forty-five patients had diffuse large B-cell lymphoma, 4 had mantle cell lymphoma, and 2 had peripheral T-cell lymphoma. The patients consisted of individuals who were not being treated and had not used steroids in the last 1 month. The patients were followed from the time of diagnosis until October 2012 in terms of life expectancy. The control group consisted of 17 healthy individuals who were not taking any medication, did not have any acute or chronic disease, and had not had fever in the last 1 week.

Patients receiving chemotherapy, using steroids in the last 1 month, or not agreeing to participate were excluded from the study. In the control group, people taking any medication, having any acute or chronic disease, or having fever in the last 1 week were excluded from the study. 

### Clinical and Laboratory Evaluations

In all cases, blood samples were taken in the morning after at least 8-12 h of fasting. Routine tests were performed immediately. For cytokine study, blood samples were again obtained in the morning after at least 8-12 h of fasting. We used empty tubes for serum and tubes containing EDTA for plasma samples. As soon as blood samples were put into tubes, they were transferred to icy media and forwarded to the laboratory within 5-10 min. Plasma and serum were separated by centrifuging at 3000 rpm and 4 °C for 10 min and were stored at -75 °C until use. All sera were then warmed to room temperature; s-IL6 and s-VEGF cytokine levels were studied with Panomics Company ProcartaTM Human Cytokine multiplex kits with the Luminex platform.

### Statistical Evaluation

For statistical evaluation of the findings of this study, SPSS 15.0 for Windows was used and p<0.05 was considered statistically significant at a 95% confidence interval. Quantitative variables were given as mean ± standard deviation. Assumptions of normality were tested with the Shapiro-Wilk test. We used parametric tests for data with normal distribution and non-parametric tests for data with non-normal distribution. The independent samples t-test and Mann-Whitney U test were used for comparison of 2 independent groups. Spearman’s correlation coefficients were used to determine the relationships between the variables. The Kaplan-Meir method was used for survival analysis. A Cox regression model was used for multivariate analysis of the parameters that were effective on prognosis.

## RESULTS

### s-VEGF and s-IL6 Levels in Patients and Healthy Controls

The median age was 52 years (min: 18, max: 90) in the patient group and 48 years (min: 40, max: 63) in the control group. The median s-VEGF levels were 11 pg/mL (min: 1.28 pg/mL, max: 159.75 pg/mL) in the patient group and 1.27 pg/mL (min: 1.00 pg/mL, max: 29.4 pg/mL) in the control group (p=0.002). The median levels of s-IL6 were 1.28 pg/mL (min: 1.10 pg/mL, max: 18.01 pg/mL) in the patient group and 1.27 pg/mL in the control group (p=0.001).

### Concentrations of s-VEGF and s-IL6 and Characteristics of Non-Hodgkin Lymphoma Patients

When the s-VEGF median value (11 pg/mL) was taken as the cut-off value, there was no difference between the groups that were above and below the cut-off for B symptoms, age, stage, erythrocyte sedimentation rate (ESR), lactate dehydrogenase (LDH) level, mortality, beta-2 microglobulin level, or IPI score (p>0.05). In patients with higher s-VEGF levels, more profound lymphopenia (p=0.034), poor performance status (p=0.027), and higher platelet levels (p=0.035) were observed. 

When the s-IL6 median value (1.28 pg/mL) was taken as the cut-off value, more advanced age, presence of B symptoms (p=0.022), advanced stage (p=0.003), poor performance status (p=0.001), high IPI score (p=0.016), increased incidence of mortality (p=0.003), more profound lymphopenia (p=0.008), higher LDH levels (p=0.006), higher platelet counts (p=0.047), and lower albumin levels (p=0.014) were observed in patients with s-IL6 levels above the cut-off.

When patients were grouped according to categories of over or below 60 years; WHO performance status of 0-1 or 2-4; serum LDH levels normal or above normal; Ann Arbor stage of I-II or III-IV; IPI score of low (L), low-intermediate (L-I), high-intermediate (H-I), or high (H); and presence of B symptoms and were compared according to median s-VEGF and s-IL6, significantly higher values of s-IL6 were only observed in patients with H-I or H IPI scores and advanced Ann Arbor staging (p<0.001 and p=0.014, respectively; Table 1).

### Correlation of s-VEGF and s-IL6 with Overall Survival in Non-Hodgkin Lymphoma Patients

**Figure 1:**
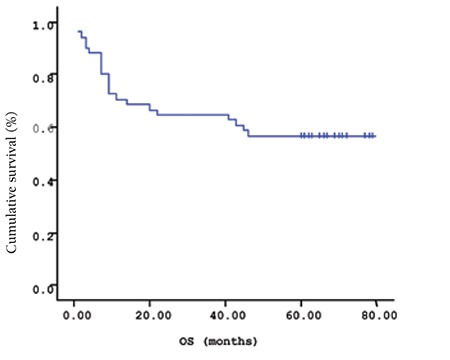
Mean overall survival (OS) based on s-VEGF levels.

**Figure 2:**
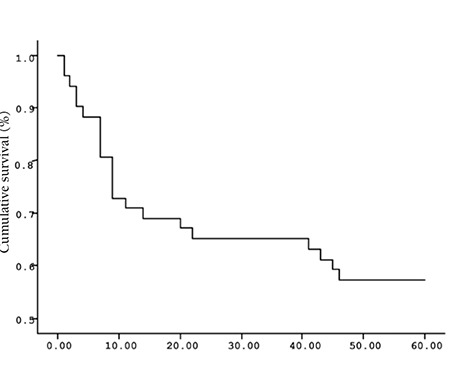
Mean overall survival (OS) based on s-IL6 levels.

**Figure 3:**
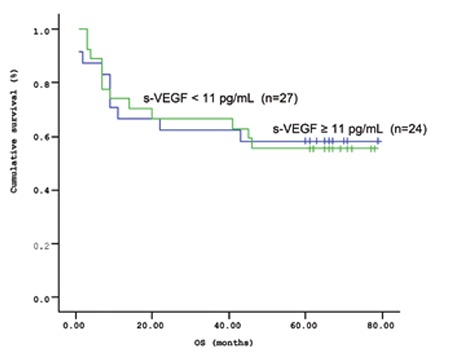
Overall survival (OS) curves of patients with lymphoma based on serum s-VEGF level (cut-off level: 11 pg/mL; p=0.926).

**Figure 4:**
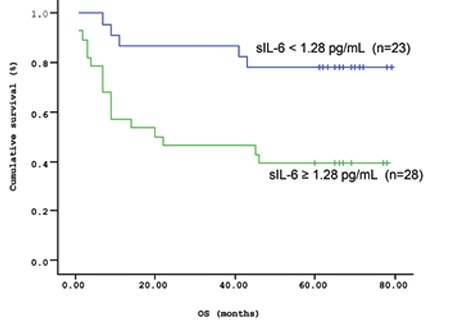
Overall survival (OS) curves of patients with lymphoma based on serum s-IL6 level (cut-off level: 1.28 pg/mL; p=0.004).

**Figure 5:**
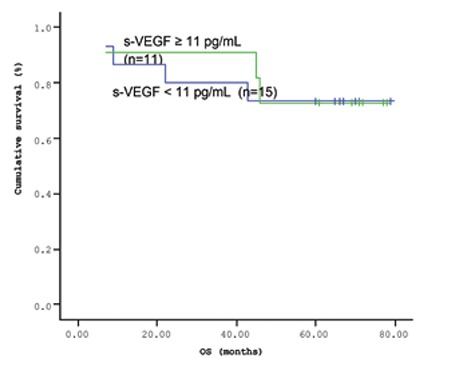
Overall survival (OS) curves of International Prognostic Index (IPI) risk group L + L-I patients with lymphoma based on serum s-VEGF level (cut-off level: 11 pg/mL; p=0.952).

**Figure 6:**
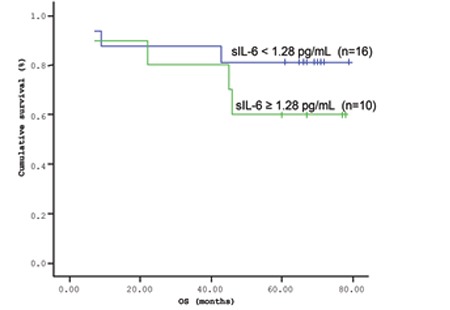
Overall survival (OS) curves of International Prognostic Index (IPI) risk group L + L-I patients with lymphoma based on serum s-IL6 level (cut-off level: 1.28 pg/mL; p=0.282).

**Figure 7:**
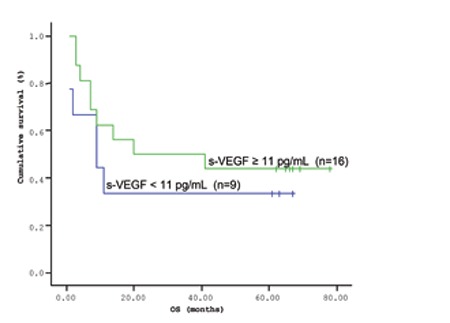
Overall survival (OS) curves of International Prognostic Index (IPI) risk group H-I + H patients with lymphoma based on serum s-VEGF level (cut-off level: 11 pg/mL; p=0.420).

**Figure 8:**
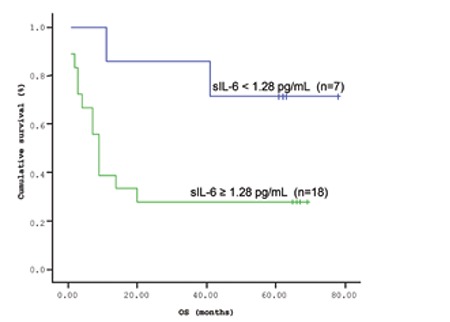
Overall survival (OS) curves of International Prognostic Index (IPI) risk group H-I + H patients with lymphoma based on the serum s-IL6 level (cut-off level: 1.28 pg/mL; p=0.035).

**Figure 9:**
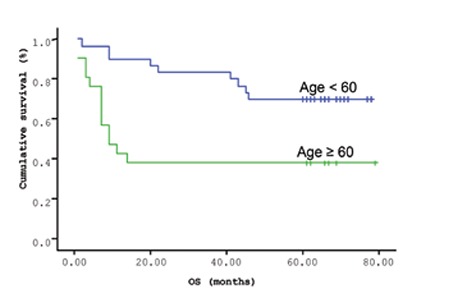
Overall survival (OS) time in patients with advanced age (≥60 years; p=0.005).

The mean survival time of the patients based on the Kaplan-Meir method was 51.9 months (Figures 1 and 2). When the cut-off value of s-VEGF was taken as 11 pg/mL, OS was 50.8 months in the group with values below the cut-off and 50.9 months in the group with values above the cut-off (p=0.926; Figure 3). When the cut-off value of s-IL6 was taken as 1.28 pg/mL, OS was 66.2 months in the group with values below the cut-off and 38.1 months in the group with values above the cut-off (p=0.004; Figure 4). When these patients were analyzed according to IPI score, no significant difference in survival was found between the patients with s-VEGF and s-IL6 values above or below the cut-off values in patients with IPI scores of L and L-I (p=0.952, p = 0.282, respectively; Figures 5 and 6). In patients with IPI scores of H-I and H, while there was no difference between the groups with s-VEGF levels above and below the cut-off (p=0.420), significantly lower survival was seen in the group of patients with s-IL6 levels above the cut-off (p=0.035; Figures 7 and 8). Survival was found shorter in patients aged ≥60 years (34 months) and longer in patients younger than 60 years (62.5 months) (p=0.005; Figure 9).

### Prognostic Factors in Multivariate Analysis of Non-Hodgkin Lymphoma Patients

Multivariate analysis performed using Cox regression analysis showed no relation between survival and s-VEGF, age, sedimentation, beta-2 microglobulin, ECOG performance, or high IPI scores (p>0.05). NHL patients with high s-IL6 levels, advanced age, elevated ESR, high beta-2 microglobulin, poor ECOG performance, and high IPI scores had a shorter duration of life and poorer prognosis. These parameters are independent risk factors in the follow-up of NHL patients (Table 2).

## DISCUSSION

s-IL6 and s-VEGF levels of NHL patients are found to be higher than those of normal healthy controls [6]. In our study, both s-IL6 and s-VEGF levels of the NHL patient group were significantly higher than those of healthy controls.

Age is an important prognostic factor for both morbidity and mortality in NHL patients. In our study, overall survival time was significantly shorter in the patient group of ≥60 years of age.

IPI score is an important prognostic marker for follow-up of patients with particularly aggressive lymphoma [8]. Recent studies showed that some prognostic factors may be included in the IPI or may be used in combination. These cytokines include s-IL6 and s-VEGF [5,6,7]. Pre-treatment levels of s-IL6 and s-VEGF are correlated with the survival of NHL patients, and both cytokines are important independent predictors of prognosis for all IPI risk groups. Disease-free survival time and OS time are shorter in NHL patient groups, especially among those with s-IL6 and s-VEGF values above the cut-off [6,9]. In our study, we found significantly shorter OS in the patient group with s-IL6 levels above the cut-off. When the IPI risk groups were divided into 2 as L + L-I and H-I + H, we found significantly shorter life expectancy and poorer prognosis in the H-I + H group patients, and especially those with s-IL6 levels above the cut-off. Unlike other studies, we did not find a difference between s-VEGF levels and the OS time or IPI risk group. As a result, s-IL6 is an independent prognostic factor that may be included in IPI risk classification.

In NHL patients, LDH levels, bulky mass, beta-2 microglobulin levels, performance status, presence of B symptoms, age, advanced stage of disease, and extranodal involvement are well-known and commonly used prognostic factors. There is usually a significant correlation among poor performance status, high LDH levels, large bulky mass, and presence of B symptoms in patients having high levels of s-IL6 and s-VEGF [6,10,11]. When the median s-VEGF value was taken as the cut-off (11 pg/mL), we did not find any differences in B symptoms, age, stage, ESR, LDH, mortality, or beta-2 microglobulin level between groups below and above the cut-off. We found more profound lymphopenia, poorer performance status, and higher platelet counts in the patient group with higher s-VEGF levels. When the median s-IL6 value was taken as the cut-off (1.28 pg/mL), we found more advanced age, more presence of B symptoms, more advanced stage, poorer performance status, higher IPI scores, increased mortality, more profound lymphopenia, higher LDH, higher platelet count, and lower serum albumin in the patient group with s-IL6 levels above the cut-off. When patients were grouped in categories of <60 years or ≥60 years, WHO performance status of 0-1 or 2-4, serum LDH levels normal or above normal, Ann Arbor stage I-II or stage III-IV, IPI score of L + L-I or H + H-I, and presence or absence of B symptoms and compared according to median s-VEGF and s-IL6 levels, we only found significantly higher s-IL6 levels in patients with H + H-I IPI scores and advanced Ann Arbor stage. We did not find any similar relationships between s-VEGF level and LDH, B symptoms, age, stage, ESR, LDH, mortality, beta-2 microglobulin level, or the value of the IPI score as were seen in the study of Xia et al. [11]. It should be kept in mind that the patient group with s-IL6 levels above the cut-off values may proceed to lymphopenia, hypoalbuminemia, advanced stage, B symptoms, poor performance status, thrombocytosis, elevated LDH, and high mortality.

Disease-free survival time and OS time are short in NHL patient groups, especially those with s-IL6 and s-VEGF values above the cut-off. These 2 cytokines can be used separately or in combination as independent prognostic factors [6,12,13,14]. In our study, we found strong correlations between s-IL6, age, ESR, beta-2 microglobulin, WHO performance status, and IPI score and OS. This shows that when high s-IL6, high ESR, high beta-2 microglobulin, poor WHO performance status, and high IPI score are detected in NHL patients, their duration of life may be predicted to be shorter. However, we could not find a difference between s-VEGF and survival due to the small patient sample.

## CONCLUSION

Patients with aggressive NHL diagnosis have higher s-VEGF and s-IL6 levels than healthy populations. In particular, if s-IL6 levels of patients are above the cut-off, OS is shorter. There was no relation between s-VEGF and OS. In addition to the s-IL6 level, age, ESR, beta-2 microglobulin, WHO performance status, and IPI score are independent prognostic factors that are effective, especially for OS, in the clinical follow-up of NHL patients.

NHL patients with high levels of s-IL6 and s-VEGF have been reported to have poor prognosis and short survival before. However, to the best of our knowledge, there is no information available for patients from a Turkish population. We think that this is the novelty of our study.

## Figures and Tables

**Table 1:**
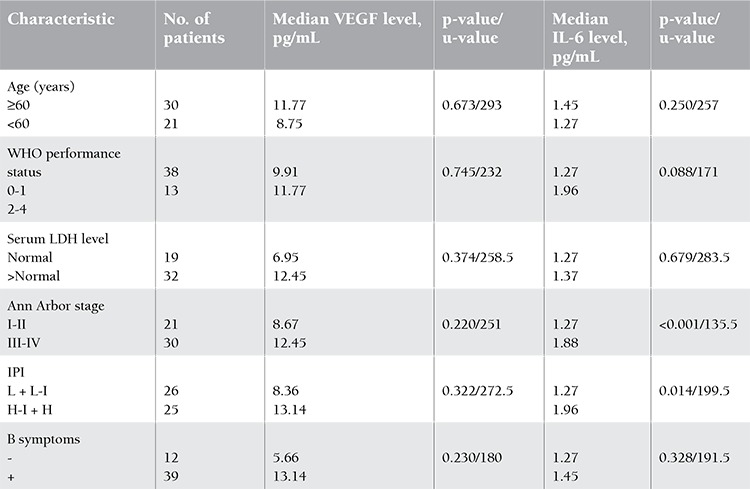
Correlation of serum levels of s-VEGF and s-IL6 with clinical prognostic factors in lymphoma.

**Table 2:**
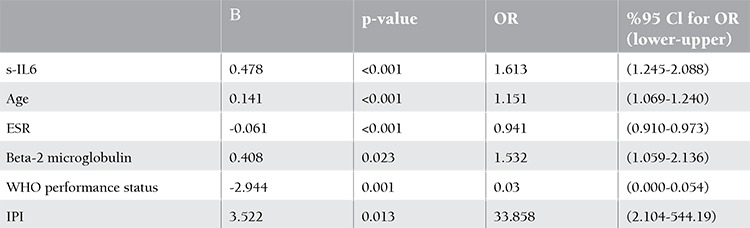
Multivariate analysis of serum s-IL6, age, erythrocyte sedimentation rate, beta-2 microglobulin, WHO performance status, and International Prognostic Index (IPI) score.
